# Embedded Wireless Sensor for In Situ Concrete Internal Relative Humidity Monitoring

**DOI:** 10.3390/s24061756

**Published:** 2024-03-08

**Authors:** Tai Ikumi, Ignasi Cairó, Jan Groeneveld, Antonio Aguado, Albert de la Fuente

**Affiliations:** 1 Smart Engineering Ltd., 08006 Barcelona, Spain; tai.ikumi@smarteng.es (T.I.); antonio.aguado@upc.edu (A.A.); 2 Department of Project and Construction Engineering, Technical University of Catalonia-BarcelonaTech, 08034 Barcelona, Spain; 3 WitekLab, 08223 Terrassa, Spain; ignasi@witeklab.com (I.C.); jgroeneveld@chatu-tech.com (J.G.); 4 Department of Civil and Environmental Engineering, Universitat Politècnica de Catalunya, 08034 Barcelona, Spain

**Keywords:** wireless sensor networks, temperature, relative humidity, monitoring, concrete

## Abstract

The moisture content within the concrete pore network significantly influences the mechanical, thermal, and durability characteristics of concrete structures. This paper introduces a novel fully embedded wireless temperature and relative humidity sensor connected to an automatic acquisition system designed for continuous concrete monitoring. Relative humidity measurements from this new sensor are compared with those obtained by a commercial system based on the borehole method at different depths (2.5 and 4.0 cm) and exposure conditions (oven drying and humid chamber). The results allow for proving that both systems provide consistent internal relative humidity measurements aligned with the exposure conditions and highlight the capability of fully embedded wireless sensors as a practical and reliable alternative to the conventional borehole method. Additionally, the continuous monitoring of the wireless cast-in sensor exhibits reliability during unintended temperature fluctuations, emphasizing the effectiveness of permanently installed sensors in promptly detecting unintended curing variations in real time. The continuous real-time information provided combined with the practicality of these sensors might assist construction managers to improve the quality control of the concrete curing process and shrinkage behavior, and ensure the integrity of concrete surface finishing.

## 1. Introduction

The amount and state of water in the concrete pore network influence the mechanical, thermal, and durability characteristics of concrete elements and structures. Proper monitoring of the water content in concrete is essential to address the following:

- *Control of the curing process*. Effective curing must ensure keeping the material under specific conditions of temperature and moisture to allow for the correct cement hydration and the development of its mechanical properties. Loss of moisture during curing can significantly slow down cement hydration as drying coarsens the pore structure of the cement paste matrix [1,2]. In fact, it has been observed that the hydration of cement paste might even stop when the relative humidity drops below about 80% [3]. This is particularly relevant in hot-weather concreting [4] and high-performance concrete applications, where low water-to-binder ratios and high binder contents are used.
- *Corrosion prevention*. Carbonation and chloride ingress rates are moisture-dependent. The progression of carbonation is the highest at intermediate moisture contents (between dry and saturated state) [5], while the diffusion of chlorides needs water in the pores to diffuse.
- *Freeze-Thaw*. The rising damp and alkali–silica reactions are aggravated by high moisture contents [6]. The prevention of these phenomena may involve the limitation of water availability, and therefore, moisture monitoring in concrete is essential to assess the effectiveness of the corrective actions.
- *Shrinkage control*. Concrete shrinks as its internal moisture content decreases, either through exchange with the environment (evaporation) or through self-desiccation (hydration of cement particles), with its magnitude proportional to the amount of moisture lost [4,7,8]. The restraint of shrinkage-induced strains caused by moisture gradients is one of the most common causes of early cracking in concrete elements. This phenomenon is particularly relevant in concrete structures with large surface areas, where water evaporation is magnified and might lead to significant shrinkage strain gradients with concrete stresses superior to its early cracking strength.
- *Concrete surface finishing quality*. When concrete is poured in slabs, water vapor migrates from the bottom to the surface to evaporate. Early applications of impermeable finishes might lead to damage associated with moisture retention such as delamination of the floor adhesive, blistering of the epoxy coating, re-emulsification of the adhesive and curling, cracking or bubbling of flooring materials. ASTM F2170-19a [9] recognizes the need to monitor relative humidity levels before the installation of floor coverings and coatings and define threshold levels [10] to avoid subsequent damages to the system [11].

The examination of the moisture condition of concrete usually involves characterization of the internal moisture content and/or the relative humidity. The first refers to the amount of water present in the concrete, typically expressed as a percentage of the total mass of the concrete. On the other hand, the latter represents the percentage of moisture in the air relative to the maximum amount it could hold at a given temperature [12]. The relationship between these two critical indicators is established by the water sorption isotherms of cement pastes, as this link relative humidity with saturation degrees [13].

The measurement of moisture content is usually established by gravimetric methods. The average moisture content (free water) is assessed by the overall weight changes on test specimens of a particular shape and size. The moisture content distribution with the depth (referred to as moisture profile) is usually assessed by sampling from a series of depths. A review of sampling techniques can be found in [14]. Despite this being a straightforward method, the sample preparation is labor-intensive and the final measured values are often affected by variability related to the slicing process [7,15]. Moreover, this method cannot measure changes in the moisture condition produced by hydration of the cement or by changes in the capillary structure of the concrete [15]. Surface-based methods are not discussed here as these are not suitable for internal humidity assessments.

Internal relative humidity measurements are a relatively cheap and reliable alternative for in situ assessment of the internal moisture state of concrete [7,11]. Nowadays, the moisture condition is usually assessed by measuring the relative humidity with an electronic RH-probe placed in a closed volume of air in contact with the material [6,8,16,17,18,19,20]. This closed volume of air can be created during casting by placing a PVC tube or sleeve into a concrete structure on site with an open end at an intended distance from the exposed surface and a rubber cork in the other end. The other alternative is to drill a hole in the hardened material and seal it with a plastic tube with an open end and a rubber cork. Readings can be obtained once hygrometric equilibrium conditions are reached in the enclosed air volume and the open concrete surface. The time required depends on the geometry, the properties of the material and the properties of the RH-probe [14]. This standardized procedure is formally outlined in ASTM F2170-19a [9], with several commercial systems available in the market, mainly oriented to the pavement construction industry (Vaisala SHM40, Proceq hygropin, Wagner Meter RH L6, amongst others).

Despite its widespread implementation, this procedure presents several limitations when it comes to the continuous long-term monitoring of concrete internal humidity. Firstly, there is a recurrent need for sealing/unsealing the closed air cavity in contact with the concrete at each measurement, which might induce disturbances in the internal humidity distribution [7]. Secondly, probe installation and placement (either drilled hole or cast-in tube) affect the concrete surface at multiple locations and constitute external objects that might interfere with the construction practices on site. Finally, the readings are manual and time-consuming (about 30 min per reading), which is not compatible with continuous monitoring.

Nowadays, there is a growing interest in full autonomous wireless embedded solutions for internal relative humidity monitoring in concrete to overcome these limitations, most of them being experimental prototypes not currently commercialized [16,17,21,22]. These solutions are based on the same principles as the aforementioned ones, as the measurements are also conducted with the use of sensors in a closed air volume in open contact with the concrete pore network. The closed air volume is integrated in the sensor body, which is kept completely embedded inside the concrete element since casting. This air cavity is protected from fresh concrete ingress through the use of a water-impermeable fabric which is permeable to vapor. This way, the air cavity is continuously sealed, guaranteeing no disruption of the humidity distribution. The humidity measurements from the sensor are localized and reflect only the depth at which the sensor is positioned. To precisely measure the moisture gradient in a concrete section, various sensors should be placed at different depths at a considerable distance from each other (to avoid disturbance of the moisture flow).

The data in such fully embedded solutions are not only securely held on the sensor inside the concrete, but data collection is also simpler and faster as there are no external units that need to be maintained. In fact, these mostly incorporate wireless automatic acquisition systems (by means of various communication systems such as LoRa, Zigbee, Bluetooth Low Energy, ISM or Wifi [23]). This allows for the autonomous and continuous recording of the relative humidity following casting, which allows for the monitoring of the progressive reductions in the internal moisture associated with concrete self-desiccation at early ages [7]. Such a high operating complexity combined with the total embedment of the sensors in concrete significantly limits the battery lifespan of this technology, being unsuitable for long-term monitoring.

Alternatively, some experimental prototypes implement passive radio frequency identification technology (RFID) oriented to reach longer service lives (required for structural health monitoring), and to reduce the complexity and economic cost of the solution [21,22]. These systems are able to communicate with the interrogator on a zero-powered backscatter mechanism but have significant limitations regarding the wireless sensing range between the tag and the reader. As a result, data collection is often compromised or impractical for continuous live concrete monitoring. Commercialized fully embedded wireless concrete sensors for the monitoring of temperature (such as [24,25,26,27,28]), distance ([29,30]), or other properties are not considered here.

Despite the recognized importance of internal moisture monitoring in concrete, there is still a lack of real experimental data openly available to support progress in both the measurement methods and the prediction of moisture in buildings [6]. This is evidenced by the lack of established general criteria among practitioners regarding common concrete drying rates and even a basic understanding of what moisture and relative humidity in concrete really represent.

The objective of this paper is to introduce a new fully embedded wireless temperature and relative humidity sensor for continuous concrete monitoring. Relative humidity measurements from this new sensor at various exposure conditions and depths are compared with those obtained from a commercial sensor based on the borehole method according to ASTM F2170-19a [9].

## 2. Materials and Methods

### 2.1. Mortar Cubes

A total of 16 cubes of 10 cm × 10 cm × 10 cm were cast to assess the internal relative humidity of mortars at 2 different depths (2.5 and 4 cm) during varying wet–dry exposure. The mortar mix adopted is specified in Table 1, which intends to simulate a typical composition of the mortar that surrounds the coarse aggregate in a conventional concrete applied in pavements. Table 2 describes the particle size distribution of the aggregates used.

The mixing procedure defined in UNE-EN 196-1:2005 [32] was adopted, where all solid components (cement, sand, and gravel) were initially dry-mixed. At the end of mixing process, the fresh mortar was poured into 10 cm × 10 cm × 10 cm molds.

### 2.2. Internal Relative Humidity Monitoring

#### 2.2.1. Borehole Method (Conforms to ASTM F2170-19a [9])

The Vaisala Structural Humidity Measurement Kit SHM40 (Vaisala, Vantaa, Finland) is adopted here to obtain reference internal relative humidity measurements of the mortar specimens, which has been designed for use with the borehole method. It uses the humidity and temperature probe HMP110 with a rugged polyurethane filled stainless steel body. The measurement range is [0, 100%RH] and [−40, +80 °C]. Reported measurement accuracy at temperatures between 0 and 40 °C is ±1.5%RH (0–90%RH) and ±2.5%RH (90–100%RH). Vaisala reports [33] errors between 5–6%RH when there is a difference of ±1 °C between the measured object and the probe at temperatures between 20 and 40 °C.

Plastic tubes (Ø17.4 mm and 120 mm length, Vaisala, Vantaa, Finland) were cast-in in 8 mortar cubes in such a way that the ends of the tubes had an open concrete surface at an intended distance from the exposed surface. Four specimens were prepared for each depth evaluated (2.5 and 4 cm). Long paper plugs were placed inside the tube to prevent the fresh mortar from blocking it. Due to the presence of aggregates, trowelling was performed to ensure a flat surface around the tube.

After 24 h, the specimens were demolded and paper plugs were removed. Then, the bottoms of the tubes were cracked with a flat-head screwdriver to help the air in the tube reach equilibrium with the humidity in the concrete. The crack between the tube and the hole was sealed with a thermo-silicon gun (Taurus Group, Oliana, Spain). Finally, the mortar dust at the bottom of the tube was cleaned with a vacuum cleaner and all tubes were sealed with a rubber plug.

Humidity measurements were initiated 3 days after the tube installation to allow for the airspace humidity to reach humidity equilibrium with the mortar, as indicated by the producer. Humidity measurements were performed by inserting the probe into the tube. Then, the tube was sealed with the rubber plug on the cable of the probe for 30 min to stabilize before starting the measurements.

#### 2.2.2. Wireless Totally Embedded Sensor

**Figure 1:**
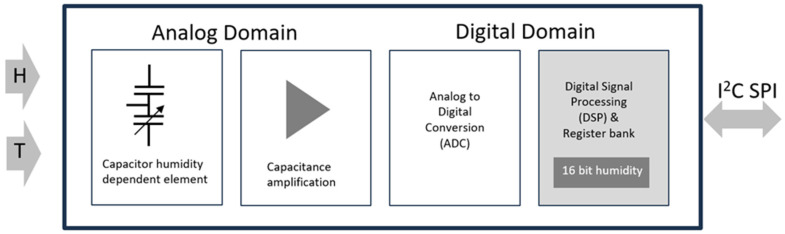
Block diagram of the sensor unit.

The new temperature and relative humidity sensor Monsec, developed by ChatuTech (Terrassa, Spain) and Smart Engineering (Barcelona, Spain), is presented and used here as a practical alternative for concrete internal relative humidity monitoring. Monsec incorporates a silicon-based integrated circuit (IC) sensible to both temperature and relative humidity with a size of 1.5 mm × 1.5 mm × 0.5 mm in contact with an airspace integrated in the body. The sensing element consists of a mixed signal application-specific integrated circuit (ASIC) that provides measurement information through the IC (also possible SPI) digital serial interface to the local microcontroller unit. Such a sensing element consists of a polymer dielectric planar structure, capable of detecting relative humidity and is manufactured using a dedicated silicon process. The digitalization of the humidity sensor is carried out in the ASIC in a digital signal processing unit (DSP). Figure 1 represents a block diagram of the sensor unit.

**Figure 2:**
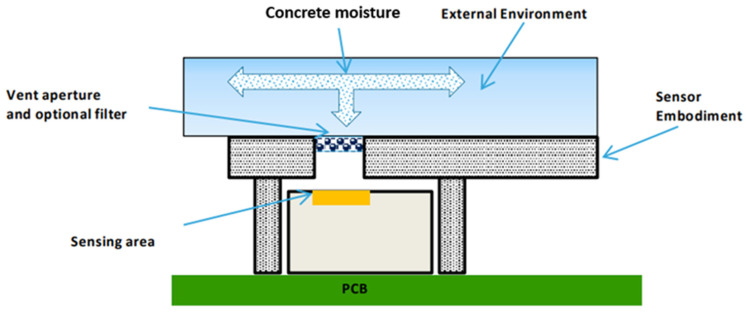
Humidity sensor system integration.

The sensor unit is integrated with the rest of the electronic system, taking special precautions to ensure that the embodiment of the sensor IC minimizes the differences between the humidity and temperature conditions of the environment under test conditions and those that represent the conditions around the sensor area. It is also important to consider the influence of heat generated by other devices close to the sensing area or due to the heating of the sensor itself. Changes in temperature are critical because these will also determine relative humidity deviations and, consequently, a slower response of the system. In this case, to improve the thermal decoupling of the sensor from the system, milling slits were created, and all unnecessary metals from the PCB around the sensor were etched. Figure 2 depicts a schematic diagram of the humidity sensor system integration.

**Figure 3:**
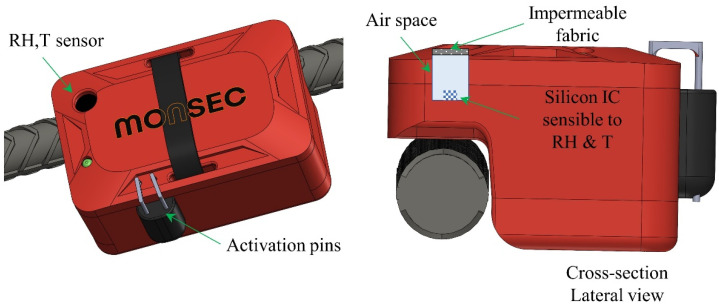
Details of the RH sensor incorporated in Monsec.

Considering the small window of the IC device exposed to the external environment and in order to obtain reliable and consistent measurements, the design is optimized to maximize sensor exposure to the external environment of concrete. This ensures a faster time response in terms of humidity and temperature. Additionally, it is crucial to guarantee that the environmental conditions match the sensing area conditions, not only in the steady state (static conditions) but also under dynamic conditions. As depicted in Figure 2 and Figure 3, the impermeable fabric located on top protects this air cavity from fresh concrete ingress while being permeable to vapor exchange with the surrounding concrete. The sensor is fully functional in condensing environments and provides an operating range of [0, 100%RH] and [−40, +125 °C]. Relative humidity and temperature accuracies reported are ±[1.5, 1.8%RH] (30–70%RH) and ±0.1 °C, respectively. For RH > 70%, the accuracy tolerance is ±[2, 3%RH].

**Figure 4:**
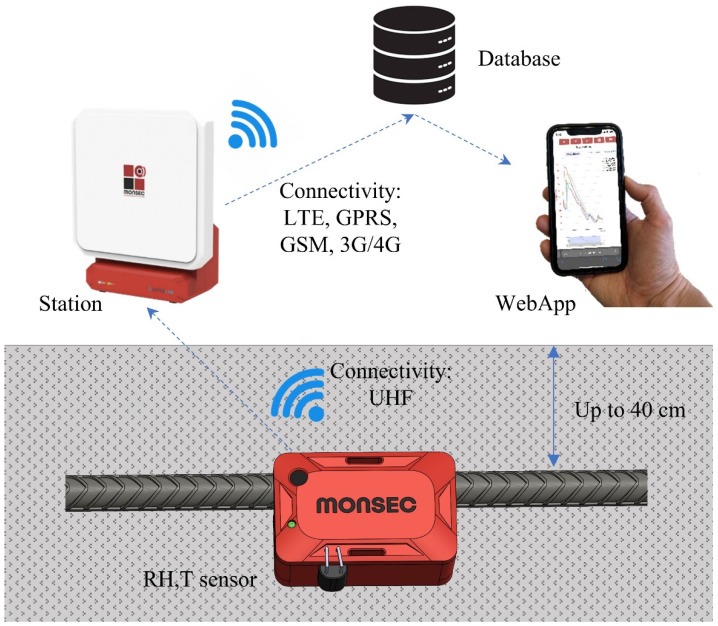
Components of the Monsec solution.

Besides the sensor element, which performs local measurements of temperature and relative humidity (Figure 3), the Monsec solution is composed of a station (a single receiver operating on the same ISM band), which wirelessly receives the recorded data and transmits it immediately to the cloud and a webApp from which measured data can be accessed. This system is entirely automated, and the receiver station can function independently using batteries and solar panels, making it a fully autonomous system without relying on the grid. The communication protocol between sensors is proprietary, secure, and exclusively compatible with Monsec sensors. Figure 4 illustrates a schematic representation of all the components involved, each necessary to achieve the wireless and remote continuous monitoring capabilities of the solution.

The wireless communication technology adopted by this sensor and the lack of accessibility to replace batteries due to complete embedment in concrete increase the cost and compromise the long-term performance of this solution. By configuring a measuring frequency of 15 min, the sensor battery lasts three months. This duration is sufficient for relative humidity monitoring during concrete setting but does not allow for the long-term monitoring of structures due to the limited battery lifespan. Additionally, the advantages in terms of practicality in installation and data collection often compensate for the higher initial cost.

**Figure 5:**
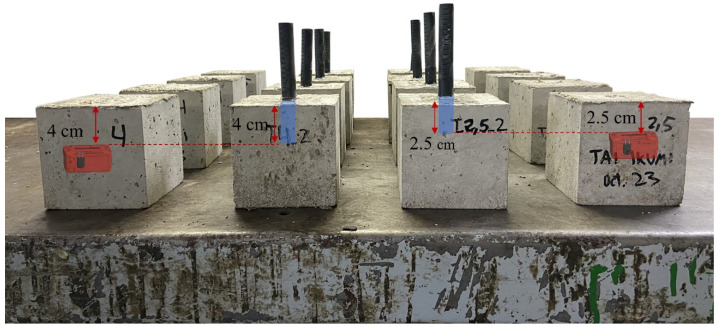
Layout of all specimens used.

The wireless sensors were placed in 8 of the freshly cast mortar specimens at the desired depths (4 sensors at 2.5 and 4 sensors at 4 cm). Measurements were initiated prior to the installation at intervals of 10 min. Figure 5 shows the final layout of the 16 specimens used, with the measuring depth for the wireless sensors and the borehole method highlighted.

### 2.3. Exposure Conditions

**Figure 6:**
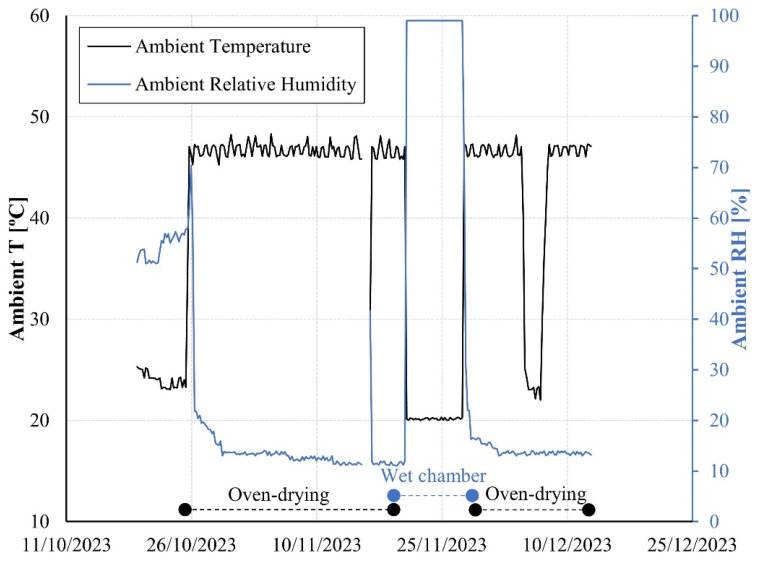
Ambient temperature over the test duration.

After demolding, the samples were exposed to dry–wet cycles to induce rapid changes in the relative humidity of concrete. The drying method adopted consisted of hot air exposure in the oven. In this method, the heat evaporates the water in the specimens and increases its vapor pressure while lowering the relative humidity of the air in the oven [14]. The constant temperature used was 47 ± 2 °C to minimize any potential damage in the pores and/or cement paste degradation [34]. The wetting cycle was performed in a humidity chamber, where all specimens were kept at 100%RH and 20 ± 2 °C. Figure 6 shows the exposed ambient temperature and relative humidity over the test duration.

## 3. Results

### 3.1. RH Measurements from the Vaisala Borehole Method

**Figure 7:**
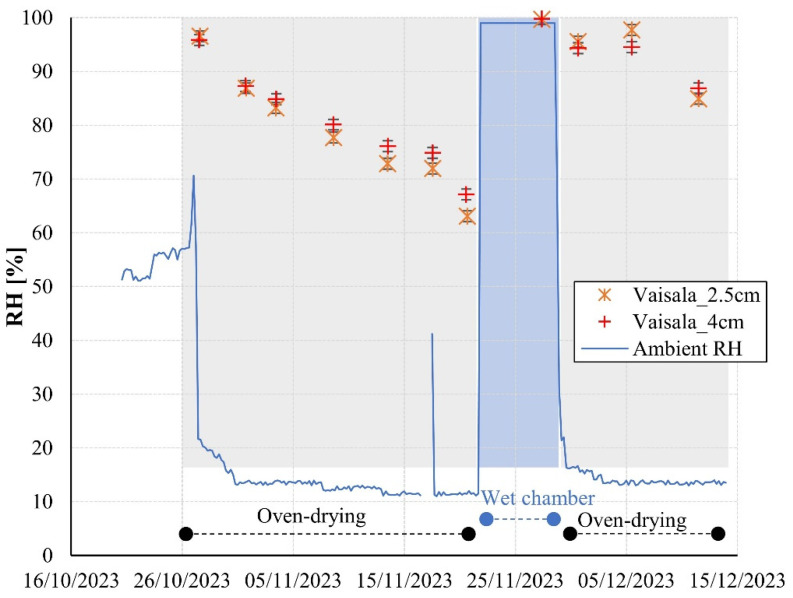
Concrete internal relative humidity at 2.5 and 4 cm measured with the borehole method.

Figure 7 depicts the ambient relative humidity and the internal relative humidity of the mortar specimens obtained through the borehole method (ASTM F2170-19a [9]) at 2.5 and 4 cm over the test duration. Relative humidities registered by the Vaisala system are presented as averages of the 4 samples evaluated and with error bars corresponding to ±1 standard deviation. As expected, mortar measurements show a gradual reduction in internal relative humidity during the oven-drying phase and a rapid increase during the wet chamber phase. Notably, the readings show minimal dispersion, with maximum coefficients of variation of only 3% among different replicates throughout the experiment.

Figure 7 illustrates that the internal moisture content of the mortar exhibits relatively minor variation across the two depths under evaluation. The average difference during the drying phase is merely 2.2%RH, and during the wetting phase, the difference is virtually negligible (<0.1%RH). The technical literature features a scarcity of studies documenting concrete relative humidity gradients in depth during the drying conditions [1,16,35,36,37,38]. Some studies indicate marginal variations in relative humidity at depths within 2.5–4 cm from the external surface in uncracked specimens [16,36], while others reveal substantial differences [1,35]. This diversity of results underscores the multitude of factors influencing moisture transport in concrete elements.

### 3.2. RH Measurements from the Wireless Embedded Sensors

**Figure 8:**
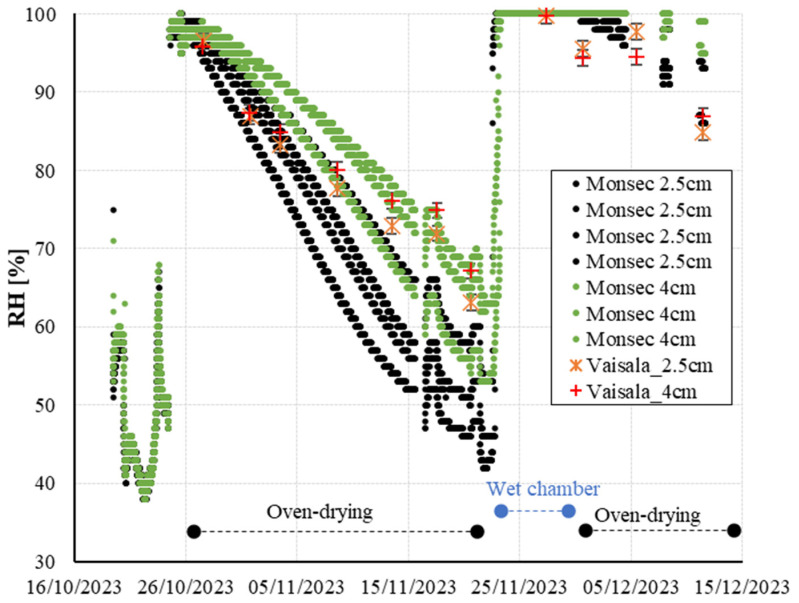
Internal relative humidity evolution in the concrete specimens at 2.5 and 4 cm.

Figure 8 shows the evolution of the mortar internal relative humidity at 2.5 and 4 cm obtained by the embedded wireless system (Monsec) and the borehole method (Vaisala). The Monsec sensors, permanently installed in the mortar, record data every 10 min. For these sensors, each data series is presented separately, as these could not be grouped under statistical criteria due to their slightly different time references.

In the pre-concreting phase, the Monsec sensors detect relative humidities ranging from 40% to 60%, aligning with laboratory conditions. Upon contact with the mortar, the humidity swiftly elevates to nearly 100%RH. Subsequently, the recorded humidity values begin a gradual decline attributed to oven-drying. As anticipated, sensors positioned closer to the surface (2.5 cm) exhibit faster drying rates compared to those at greater depths (4 cm). Approximately two weeks later, the relative humidity initiates a stabilization phase, with values between 40% and 60% RH. During this period, the cast-in sensors show a significant dispersion within specimens of the same depth (2.5 and 4 cm). This might be explained by slight sensor position variations in the vertical direction (significant in regions with large moisture gradients) [39] and/or differences on the characteristics of the material (components and pore network) around the sensor location.

Following one month of oven-drying, the samples undergo transfer to a wet chamber, triggering a rapid increase in the internal humidity of the mortar as detected by all sensors, reaching values close to 100%RH. Subsequently, after a 4-day interval, the samples are reintroduced to the oven under identical drying conditions as the initial phase. At this stage, a gradual reduction in the relative humidity of the mortar is once again observed, with more pronounced effects noted in the sensors situated at the outermost layers. Unfortunately, complete drying curves could not be completed due to limitations in the sensor’s internal battery. With a measurement frequency of 10 min, the sensor signals were lost approximately 50 days post-activation.

### 3.3. RH Measurements from the Borehole Method vs. Wireless Cast-In Sensors

As depicted in Figure 8, both the cast-in sensors and the borehole method yield internal relative humidity measurements of the mortar that align with the adopted exposure conditions. Throughout the initial drying cycle, the average difference between measurements from both systems at a depth of 4 cm amounts to 3.4%RH. In the more superficial layers (2.5 cm), the average difference increases to 6.7%RH. These disparities cannot be directly ascribed to inaccuracies in the precision of either system’s readings, as potential variations in the exact positioning of the sensors/tubes among the various test specimens considered might have played a significant role.

Additionally, the discrepancy observed between the two systems might not be attributed to the difference in the size of the air chamber where the relative humidity of the concrete is measured (significantly larger in the borehole method). Previous studies reported negligible influence in the size of the macro-pore inside which the embedded RH sensor was inserted [7]. This is attributed to the quantity of water that is necessary to shift the humidity of the macro-pore being very low in comparison to the quantity of water that the cementitious matrix can release upon drying [7]. However, it is essential to acknowledge that the studies have not assessed air chambers as small like the one in the Monsec system, making it impossible to dismiss its potential influence.

Another potential factor that could account for the reported measurement discrepancies relates to the disturbance of moisture flow around each system [14,39]. The distinct sizes and shapes of the two systems may lead to variations in moisture conditions in close proximity to the sensors. Nilsson and Fredlund [39] quantified this phenomenon through experimental and numerical analysis for various geometries and orientations, reporting differences of up to 10%RH in larger width probes. Additional research is needed to assess the influence of both sensor bodies on the moisture flow and how this might affect the reliability of the measurements.

### 3.4. Temperature Variation Effects

**Figure 9:**
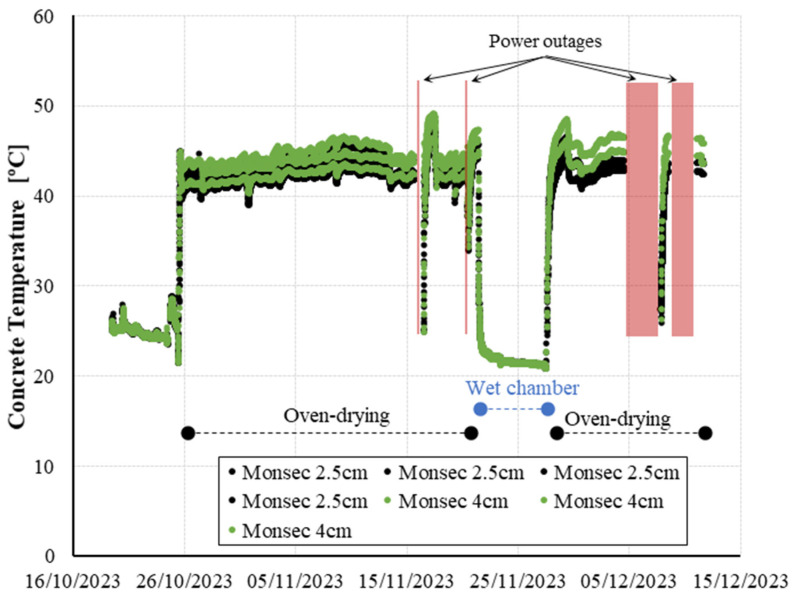
Internal concrete temperature evolution measured by Monsec sensors.

Figure 8 shows RH peaks at some specific dates that deviate from the overall trends previously described. These deviations are directly linked to variations in the exposure temperature conditions, as temperature plays a key role in moisture measurement [40,41]. In Figure 9, the internal temperature of the concrete, as recorded by the same sensors, reveals instances or intervals where the power supply from the laboratory was interrupted. This interruption led to a shift in exposure temperature, as the oven temperature equilibrated with the laboratory’s ambient temperature (approximately 20 °C). Upon resumption of power, the furnace was reactivated, initiating a progressive heating of the specimens until the previous temperature was reestablished.

This inadvertent thermal fluctuation accounts for the atypical relative humidity readings recorded within the specimens on 17 and 21 November. These peaks align precisely with the furnace reactivation and the ensuing temperature increase, which underscores the efficacy of continuous monitoring sensors in promptly detecting unintended temperature variations in real time. Previous studies also reported reliable measurement results during temperature variations for cast-in sensors as this method ensures no temperature difference between the sensor and concrete [14]. Jensen and Hansen [42] proposed that a temperature difference of 1 °C between the sensor and the concrete material might introduce an error of approximately 6%RH.

Furthermore, examining the relative humidity evolution during these periods of furnace interruption/reactivation reveals interesting trends. It can be observed that a positive correlation between the rise in concrete temperature and the increase in measured humidity within the concrete. This contradicts the inverse correlation established between relative humidity and ambient temperature in open-air conditions, where an increase in temperature results in a decrease in relative humidity. This distinctive behavior in concrete aligns with findings reported by other researchers, indicating the impact of temperature on the vaporization of capillary water bound in the pores and the resulting water vapor transfer in capillary passages between the pores [14,17].

## 4. Conclusions

This paper presents a novel fully embedded wireless temperature and relative humidity sensor for continuous concrete monitoring. Relative humidity measurements from this new sensor at various exposure conditions and depths are compared with those obtained from a commercial sensor based on the borehole method. The following outlines can be concluded:

- The comparison at various depths and exposure conditions indicates that both systems yield consistent internal relative humidity measurements aligned with the adopted conditions. These results highlight the capability of fully embedded wireless sensors as a practical and reliable alternative to conventional methods.
- The wireless cast-in sensor method has given reliable relative humidity measurements during unintended temperature variations, as this method ensures no temperature difference between the sensor and the concrete. This emphasizes the efficacy of permanently installed sensors over discrete monitoring in promptly detecting unintended curing variations in real time.
- Further research is needed to assess the influence of moisture flow disturbance around the cast-in sensor body and how this might affect the reliability of the measurements.

## Figures and Tables

**Table 1:** Mortar mix evaluated [31].

Material	[kg/m^3^]
CEM II/A-L 42.5 N (Promsa, Barcelona, Spain)	330
Coarse agg. 4/10 mm (Promsa, Barcelona, Spain)	260
Sand 0/4 mm (Promsa, Barcelona, Spain)	1543
Water	165
Water/cement ratio	0.50
Master Ease 3850 Superplasticiser	0.90% by cement weight
Master Pozzolith 7003 Plasticiser	0.18% by cement weight

**Table 2:** Aggregate grading [31].

Sieve Size [mm]	4–10	0–4
	[% Passing]	
40	100.0	100.0
20	100.0	100.0
10	96.1	100.0
4	0.8	99.9
2	0.40	83.3
1	0.4	52.1
0.5	0.4	33.7
0.25	0.4	22.4
0.125	0.4	17.6
0.063	0.4	14.9

## Data Availability

The data presented in this study are available on request from the corresponding author.
